# The Role of the Complement System in Synaptic Pruning after Stroke

**DOI:** 10.14336/AD.2024.0373

**Published:** 2025-06-25

**Authors:** Hongying Liu, Min Jiang, Zhiying Chen, Chuan Li, Xiaoping Yin, Xiaorong Zhang, Moxin Wu

**Affiliations:** ^1^Department of Medical Laboratory, Affiliated Hospital of Jiujiang University, Jiujiang, 332000, China.; ^2^Jiujiang Clinical Precision Medicine Research Center, Jiujiang, 332000, China.; ^3^Department of Neurology, Affiliated Hospital of Jiujiang University, Jiujiang 332000, China.

**Keywords:** complement, microglia, stroke, synaptic pruning

## Abstract

Stroke is a serious disease that can lead to local neurological dysfunction and cause great harm to the patient’s health due to blood cerebral circulation disorder. Synaptic pruning is critical for the normal development of the human brain, which makes the synaptic circuit completer and more efficient by removing redundant synapses. The complement system is considered a key player in synaptic loss and cognitive impairment in neurodegenerative disease. After stroke, the complement system is over-activated and complement proteins can be labeled on synapses. Microglia and astrocytes can recognize and engulf synapses through corresponding complement receptors. Complement-mediated excessive synaptic pruning can cause post-stroke cognitive impairment (PSCI) and secondary brain damage. This review summarizes the latest progress of complement-mediated synaptic pruning after stroke and the potential mechanisms. Targeting complement-mediated synaptic pruning may be essential for exploring therapeutic strategies for secondary brain injury (SBI) and neurological dysfunction after stroke.

## Introduction

Stroke is an acute cerebrovascular disease that causes brain tissue damage due to the blockage or sudden rupture of blood vessels in the brain. Its disability and mortality are particularly high worldwide [1]. The epidemiological data show that the incidence rate of stroke in China increased by 86% in 2019 compared with 1990, mainly due to the aging population and tread of younger onset in recent decades [2, 3]. In addition, economic development, urbanization and the risk factors such as hypertension, diabetes, smoking, excess alcohol consumption and high-fat diets are closely related to it [4-6]. It poses a substantial economic burden on both society and families. According to the different etiology and pathogenesis, stroke can be divided into ischemic and hemorrhagic stroke (IS and HS). IS is caused by blockage or severe stenosis of cerebral vascular, leading to impaired blood circulation and reduced perfusion, engendering an ischemic core, surrounded by a hypoperfused region termed the penumbra [7]. HS is due to the sudden rupture of blood vessels in the brain, resulting in a large number of blood gathered together to form a hematoma, which in turn compresses the brain and causes structural damage [8]. Neurons affected by initial ischemic and hematoma insult trigger a rapid cascade of events, including cellular excitotoxicity, calcium overload, mitochondrial disturbance, oxidative stress, neuroinflammation, blood-brain barrier (BBB) impairment and neuronal cell death, all of which promote secondary brain injury (SBI) [9-11]. Damaged neurons release damage-associated molecular patterns (DAMPs), activate microglia and astrocytes, and then release pro-inflammatory cytokines and chemokines, thereby recruiting peripheral immune cells such as monocytes, T cells, and neutrophils to injury area [7]. Similarly, endothelial cells will also be activated, enhance the expression of adhesion molecules, and promote cytokines and chemokines production. The chemokines recruit white blood cells (WBC) and complement components to the site of injury, further amplifying the inflammatory response and aggravating brain damage [12]. In addition, the activation of thrombin and lysis of erythrocytes further aggravates SBI following HS [13]. Blood vessel rupture will release a variety of components including red blood cells (RBC), WBC, platelets, and hemoglobin into the brain parenchyma and cause inflammation, oxidative stress, and cytotoxicity by releasing various inflammatory factors, chemokines, free radicals, and other toxic substances, further destroying the BBB and aggravating brain edema and neuron damage [14-16]. Revealing the cellular and molecular mechanisms after stroke are also the key directions for the treatment. The key to stroke treatment and recovery is getting to the hospital quickly. For IS, clinical intervention such as intravenous thrombolysis using tissue plasminogen activator (tPA) and mechanical thrombectomy contribute to the recanalization of cerebral vessels [17]. Although antithrombotic therapies such as anticoagulant and antiplatelet drugs are recommended for almost all patients without contraindications, pharmacology remains limited, suggesting the need to explore new therapeutic strategies for IS. On the other hand, the treatment for HS depends on what part of the brain is bleeding and how much. Emergency treatment for HS focuses on controlling the bleeding and reducing intracranial hypertension. If the area of bleeding is large, surgery is required to remove the blood and relieve pressure, and repair of blood vessel damage including aneurysm clipping, coil embolization and stereotactic radiosurgery [18]. Although the treatment of stroke has made advanced progress in recent years, many challenges remain, including lack of effective anti-inflammatory medications and how to ameliorate post-stroke dementia. In addition, the selection and implementation of rehabilitation therapy must also take into account the specific etiology of stroke and individual differences to develop an appropriate rehabilitation program. The first 4.5 hours after stroke onset is the golden time for treatment, and if left untreated, there are nearly 1.9 million neurons, approximately 14 billion synapses and 7.5 miles of nerve fibers lost every minute [19], which contributes to neuronal dysfunction. Typically, patients with stroke have a poor prognosis, with high proportion experiencing sequelae such as disability, cognitive impairment, and depression [20]. In addition, it has been reported that more than one-third of stroke survivors may suffer from post-stroke cognitive impairment (PSCI) [21, 22].

PSCI is a common complication characterized by cognitive impairment in the 3-6 months after stroke [23]. Mini-mental state examination (MMSE) and montreal cognitive assessment (MoCA) are the most commonly used screening tools for cognitive impairment after stroke [24, 25]. Current data suggest that 25-30% of IS survivors experience immediate or delayed vascular cognitive impairment (VCI) or vascular dementia (VaD) [26]. HS also cause damage to brain regions such as executive function, behavior function, and memory function, leading to cognitive decline [27]. The causes of cognitive dysfunction include neuronal death [28], demyelination [29], and synapse loss [30, 31]. Synapses, also known as neuronal junctions, play a critical role in facilitating the transmission of electric nerve impulses between two neurons or between a neuron and an effector cell such as a gland or muscle cell. It has high plasticity to regulate information transfer between neurons. Through synaptic transmission, the brain can achieve advanced functions such as learning, memory, and cognition. Proper synaptic connections form neural circuits that serve as the basis for human and animal behavior. Synaptic dysfunction closely relates to a variety of neurological diseases, which is not only an important pathological manifestation in neurodegenerative diseases (NDDs), but also the main cause of PSCI. After IS, the entire or specific regions of the brain suffer from ischemia and hypoxia, leading to neuronal and synaptic damage. Evidence shown that within one month after IS, the synaptic density of the ischemic lesion and peri-ischemic area further decreased with the passage of time [32, 33]. In the middle cerebral artery occlusion (MCAO) model rat, the expression of synaptic marker synapsin I and postsynaptic marker postsynaptic density protein 95 (PSD95) are decreased, and the density of dendritic spines are reduced by Golgi-Cox staining [34]. Similarly, synapse loss is also observed in patients with intracerebral hemorrhage (ICH) [35]. Guo et al [36] showed that significant synapse loss at day 1 after ICH by transmission electron microscopy (TEM) in an autologous blood-induced ICH model rat. Li et al [37] found significant synaptic loss at day 3 and Nguyen et al [38] examined the average dendritic length around the hematoma at days 7 and 60 after ICH and found that the dendrites atrophy at day 7 in collagenase-induced ICH model rat. Another study suggested that PSD95 protein levels was significant decreased in cortical neurons in perihematoma at 6 hours, reaching a minimum at 12 hours and leading to synaptic dysfunction in collagenase and autologous blood induced ICH model rat [39]. These results suggest that there is synaptic loss after stroke, which may be one of the key causes of brain injury and PSCI.

Synaptic pruning is an important physiological phenomenon in the development of the central nervous system (CNS). Mounting evidence has shown that glial cells participate in synaptic pruning through a variety of signal pathways, such as complement proteins, phosphatidylserine (PS), C-X3-C motif chemokine receptor 1 (CX3CR1) [40], among which the complement system is one of the most classic pathways. The complement system is an important part of the innate immune system and consists of a group of secreted and membrane proteins that play a critical role in combating pathogens in the CNS. Overactivation of the complement system is associated with inflammatory responses in a variety of diseases, such as multiple sclerosis (MS), Alzheimer’s disease (AD), stroke, and traumatic brain injury (TBI) [41]. The complement cascade can be activated to form a membrane attack complex (MAC), which leads to neuroinflammation, brain edema, and neuronal and synaptic loss, thereby causing SBI and affecting the recovery of neurological impairment [42]. In addition, excessive complement accumulation can lead to abnormally activated microglia and astrocytes devouring synapses, resulting in synapse loss and neuronal cell death [43-45]. In this review, we summarize current understanding of complement-mediated synapse loss and explore possible treatment strategies for brain injury after stroke.

## Overview of synaptic pruning and glia-mediated synaptic pruning

As the CNS and peripheral nervous system (PNS) develop and mature, the brain forms redundant neuronal connections. Those infrequently used connections will be engulfed by microglia or astrocytes to reach an appropriate number of synapses to refine the synaptic circuit, a process termed as synaptic pruning [46]. Synaptic pruning is also a fundamental process which enhances learning and memory function [47].

## Glia-mediated synaptic pruning under physiological conditions

Microglia are resident macrophages of the brain and mainly in a resting state and constantly monitor the surrounding microenvironment under normal physiological conditions. It modulates brain function by engulfing dead neuronal debris, synapses, and producing cytokines [48-50]. Microglia-mediated synaptic pruning plays a critical role during early brain development and is first observed in the developing hippocampus [51]. Some complement molecules mark synapses as “eat me” signals, prompting microglia to recognize and engulf them [40]. Astrocytes are the most abundant glial cells and play a vital role in maintaining physiological homeostasis such as synaptic function, BBB maintenance, and neuronal metabolism in the CNS [52]. Astrocytes recognize “eat me” signals through the two phagocyte receptors, multiple-EGF like domains 10 (MEGF10) and mer tyrosine kinase (MERTK) on their surface, which mediate synaptic pruning in the CNS [53, 54]. In addition, Lee et al [55] found that the clearance of excitatory synapses by astrocytes is inhibited and the degree of synaptic damage is increased in MEGF10 knockout mice, eventually showing long-term synaptic plasticity defects and hippocampal memory formation disorders.

## Glia-mediated synaptic pruning after stroke

When pathologically damaged, microglia actively trim or remove damaged or redundant synapses to maintain normal synaptic function [56, 57]. Microglia are activated within hours and proinflammatory factors, such as TNF-α, IL-1β, IL-6, IFN-γ, chemokines, nitric oxide (NO), reactive oxygen species (ROS), and matrix metalloproteinase-9 (MMP-9) are released in response synapse loss after stroke, which aggravate neuronal death and disrupt the BBB [12, 58-60]. Microglia-mediated phagocytosis is generally essential for synaptic debris removal and brain recovery, but may also cause damage to the neuronal and synaptic structure after stroke and other diseases [61-63]. Synaptic fragment labeling has been detected in microglia after stroke brains [64]. Microglia phagocytose viable neurons during the acute phase of IS, and suppression this process prevents loss of functional neurons and death [62]. A study found that inhibiting the stimulator of interference genes STING, a key participant in innate immune response, can suppress the phagocytic ability of microglia against stroke-affected synapses and promote the recovery of synaptic density [65]. Genetic blockade of microglial Na/H exchanger (NHE1) promotes the improvement of dendritic spine plasticity and cognitive recovery after stroke [66]. Recently, Alawieh et al [67] and Wu et al [68] found that microglia mediate synaptic pruning via the complement pathway, leading to decreased synaptic density and cognitive decline. Inhibiting complement overactivation or microglia phagocytosis rescues synaptic loss, attenuates brain damage, and improves neurobehavior in stroke mice model. The role of astrocytes is similar to that of microglia after stroke. Each astrocyte occupies a separate and non-overlapping region in the brain, interacting with synapses to form a precise and complex network. However, stroke disrupts this relatively independent relationship, and synaptic debris is detected in astrocytes [69]. Emerging studies suggest that newly formed synapses can be engulfed by reactive astrocytes (RAs), a form of neurotoxicity [70]. RAs produce and release a large number of inflammatory mediators (TNF-α, IL-1α, IFN-γ) and freer radicals, directly or indirectly induce neuroinflammation, leading to·neuronal apoptosis or necrosis [71, 72]. On the one hand, astrocytes are capable of expressing substances thrombospondin 1 and 2 (TSP-1/2) and exert a protective effect on synapses in focal cerebral ischemia model mouse [73]. However, they are also capable of phagocytosing synapses, thereby inhibiting the recovery of neurological function following stroke. Luo et al [74] found that thrombin was detected on the membrane of astrocytes, which activated the Tab/NF-kB signaling pathway and then initiated injury. Morizawa et al [69] discover that phagocytic astrocytes up-regulate ATP-binding cassette transporter (ABCA1) and its pathway molecules MEGF10 and engulfment adapter phosphotyrosine-binding domain containing 1 (GULP1), thereby engulfing degraded neuronal and synaptic debris, myelin and immune cell debris in MCAO model mice (Fig. 2). Disruption of ABCA1 in RAs reduces astrocyte phagocytosis and improves neurobehavioral outcomes. Shi et al [61] recently found that in the MCAO and collagenase-induced ICH model mice, both microglia and astrocytes engulf synaptic elements, including presynaptic (SYP) and postsynaptic (Homer-1) proteins. Moreover, the expression levels of MEGF10 and MERTK in microglia and astrocytes significantly increased at day 14, indicating an enhanced ability to phagocytose synapses (Fig. 2). In addition, comparative transcriptomic analysis of IS and HS showed that astrocytes are significant differences in the expression and biological processes of genes related to phagocytosis. In IS, glial phagocytosis can be inhibited by specifically knocking out MEGF10 and MERTK both microglia and astrocyte phagocytosis receptors, thereby improving neurobehavioral outcomes and alleviating brain injury. However, in HS, only inhibition of microglia-mediated phagocytosis can improve neurobehavioral outcomes. In conclusion, complement system-mediated synaptic pruning in glia is one of the most studied pathways, the role of the complement system and complement-mediated synaptic pruning after stroke will be outlined next.

**Figure 1.**
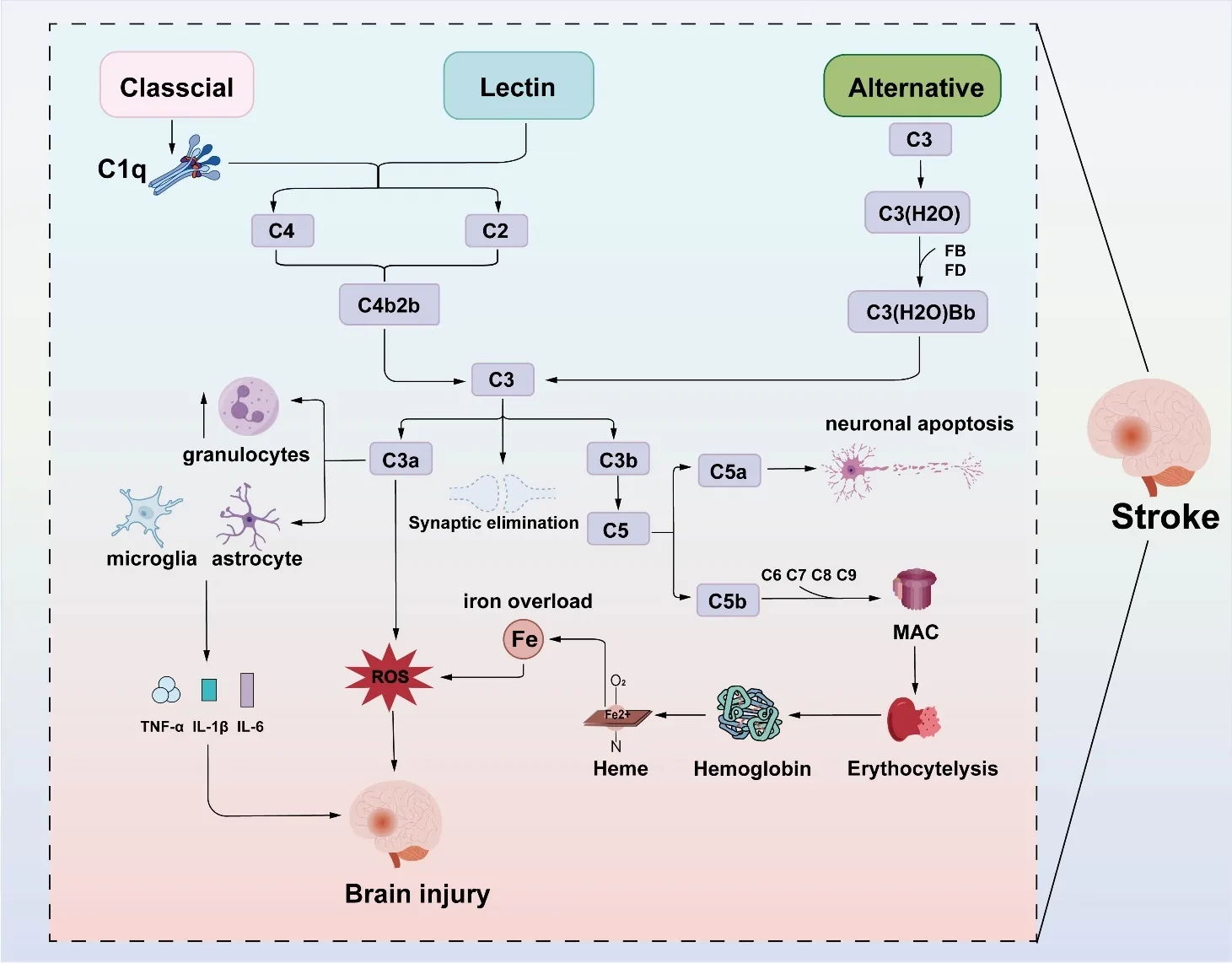
**Schematic diagram of complement-related mechanisms after stroke**. Complement is activated through three proximal pathways, promoting inflammation and MAC generation. C3 can mediate synaptic elimination. It is also decomposed into C3a and C3b by C3 invertase. C3a can activate microglia and astrocytes to produce pro-inflammatory factors, causing brain injury. C5 decomposes into C5a and C5b by C5 convertase. C5a can cause neuronal apoptosis in IS. C5b attracts downstream complement proteins C6-C9 to form MAC, leading to RBC lysis, release of hemoglobin and its degradation product iron, and causing brain injury in ICH.

## Overview of the complement system and its role after stroke

### Complement cascade overview

The complement system, also known as the complement cascade, plays a crucial role in innate immunity by rapidly recognizing and removing pathogens, cell debris, and misfolded proteins, playing a crucial role in host defense and tissue homeostasis [75, 76]. The complement cascade, involving 30 serum and membrane-bound proteins [77], is activated through the classical, lectin, and alternative pathways that converge at the site of C3 convertase.

The classical pathway (CP) is activated when an activator binds to the C1 complex, which is composed of C1q, proenzymes C1r2 and C1s2. Activators can be immunoglobulin of the Fc domain of antibodies in immune complexes, or non-immunoglobulin activators such as apoptotic cell debris, proteins, and other polyanionic substances [78]. The C1 complex cleaves components C4 and C2, generating C4a, C4b and C2a, C2b, respectively. C4b and C2b form C3 convertase (C4b2a). The lectin pathway (LP) activation is very similar to that of CP, except that the recognition pathway, which is initiated by the binding of mannose-binding lectin (MBL) or ficolin to mannose on the surface of pathogens [79], followed by the activation of MBL-related serine proteases MBL-associated serine protease 1 (MASP-1) and MBL-associated serine protease 2 (MASP-2), cleavage C4 and C2 to form C3 convertase. The alternative pathway (AP) can be activated by spontaneous hydrolysis and acts as an amplification loop for other pathways [80]. It is produced by the hydrolysis of the unstable internal thioester bond in C3 in the plasma to produce C3(H_2_O), which is subsequently activated, and in turn, C3(H_2_O) binds to factor B (FB) and is cleaved by factor D (FD) to form C3 convertase. C3 convertase will cleave C3 to produce C3b, which can combine with FB to form the proconvertase C3bB complex, which will be decomposed by FD to form the active but unstable C3 convertase [81, 82].

In the complement reaction, the C3 converting enzyme plays a crucial role by breaking down C3 into C3a and C3b. C3a is an anaphylatoxin that can trigger complement-mediated inflammatory responses. C3b can be cracked as iC3b under the action of H factor and I factor, then combined with C3 convertase to form C5 convertase (C4b2b3b or C3bBb3b) [78], so that C5 can be cleaved into C5a and C5b. C5b deposits on the cell surface and attracts the downstream complement proteins C6-C9 to form MAC (Fig. 1) [83].

## The role of complement after stroke

In 2004, Pedersen and colleagues found that transient systemic complement activation occurs after acute ischemic stroke (AIS) in humans patients [84]. Mocco et al [85] found that C1q, C3, and C5 deposit in the ischemic area of the ipsilateral cerebral hemisphere after 24 hours in the MCAO model mice. C3a is a 9 kDa allergenic peptide derived from C3. It exerts its function by binding to the specific C3a receptor (C3aR) on effector cells [85, 86]. Zhao et al [87] found that the C3aR is highly expressed in ischemic brain tissue and endothelial cells, and can significantly enhance the permeability of endothelial cells in cell culture. A recent study suggested that C3a attenuated astrocyte reactivity, stimulated global white matter reorganization, increased peri-infarct structural connectivity, and accelerated motor recovery [88]. C5a, a protein composed of 74 amino acids and one of the products of C5 cleavage, is an important inflammatory mediator to brain injury in IS [89, 90]. C5a showed delayed elevation 7-14 d after human with IS [91]. In the middle cerebral ischemia/reperfusion (MCAO/R) model rat, C5a is significantly overexpressed in the ischemic penumbra [92]. The production of C5a can cause neuronal apoptosis (Fig. 1) [93]. Kim et al [94] found that the use of C5a receptor antagonists (C5aRA), a cyclic hexapeptide AcF, significantly reduces infarct volume and improves neurological function in transient MCAO (tMCAO) model mice. The inhibition of complement also promotes angiogenesis and tissue repair after AIS. In the tMCAO model mice, CR2 inhibitors CR2-fH can up-regulate nerve growth factor (NGF) and vascular endothelial growth factor (VEGF) expression, enhance neuronal migration and improve neurological deficits and motor function [95].

Complement is also activated in the early stage of ICH. In 2000, Hua et al [96] found that complement cascade activation and MAC formation occur in the perihematomal area at 24 and 72 hours in autologous blood-induced ICH model rats. Anaphylatoxins C3a and C5a can cause chemotaxis of neutrophils and mast cells, thereby enhancing neuroinflammation, causing increased capillary permeability, and aggravating cerebral edema [90, 97, 98]. C3a also induces rapid activation of perihematomal microglia and promote the secretion of inflammatory factors such as TNF-α, IL-6 and IL-1β, thus aggravating neuroinflammation (Fig. 1) [99]. C5a stimulates the release of neutrophil-associated proteases, contributes to the degradation of VE-cadherin (an adhesion junction protein) and endothelial barrier dysfunction, and damages vascular permeability [100]. C5a/C5aR promotes the expression of fibrinogen-like protein 2 (Fgl-2) (Key enzymes in the coagulation cascade) in the perihematomal area by activating extracellular signal-regulated kinase (EPK1/2) and p38 mitogen-activated protein kinase (p38 MAPK), aggravating brain injury and neurological dysfunction [101]. MAC causes RBC lysis and releases hemoglobin and its degradation product iron, leading to ROS generation and brain damage (Fig. 1) [102-105].

In short, the excessive activation of the complement system after stroke can lead to the aggravation of neuroinflammation, oxidative stress, cellular lysis, neuronal death, and other pathological mechanisms, thereby aggravating brain injury. Emerging evidence revealed that the activation of the complement pathway leads to aberrant activation of synaptic pruning by microglia, and ultimately leading to synaptic loss and neuronal cell death in TBI, tMCAO and neonatal hypoxic-ischemic (HI) model mice [44, 67, 68, 106]. Furthermore, normobaric hyperoxia (NBO) therapy [68], endothelial cell transplantation [107] and hydrogen-rich saline (HS) [106] can mitigate complement-mediated synaptic pruning and protect against stroke, which may provide new therapeutic strategies in further. Therefore, it is crucial to focus on the mechanisms by which the complement system mediates synaptic pruning in neurological diseases and stroke.

## The role of the complement system in synaptic pruning in neurodegeneration disease

NDDs are a group of heterogeneous neurological diseases that lead to progressive loss of neurons in the CNS or PNS, which affect millions of people worldwide [108]. During neurodevelopment, the complement cascade is involved in a variety of processes, including neural tube closure, neural progenitor cell proliferation and differentiation, neuronal migration, and synaptic pruning. Various NDDs are considered to be involved in synaptic loss in the early stage [109, 110]. Mounting evidence has shown that complement cascades are significantly upregulated in AD, glaucoma, and other NDDs [111-113].

C1q plays a crucial role in synaptic pruning during the developmental stages of the CNS. Stevens et al [114] demonstrated that C1q locates on adult retinal synapses before the synaptic loss and postnatal retinal ganglion cells (RGC) death in response to astrocytes in an animal model of glaucoma. Subsequently, astrocytes recognize and participate in synaptic pruning, while mice lacking C1q have defects in synaptic pruning. Similarly, in J20 transgenic model mice, oligomeric amyloid-β (oAβ) increases the C1q level on synapses in a region-specific manner, triggering the downstream CP and enhancing microglial phagocytosis of synapses. Inhibitions of C1q, C3 or CR3 reduce the number of phagocytic microglia and the degree of synaptic loss [45]. Similarly, in the peritoneal contamination and infection (PCI)-induced sepsis model mice, C1q labeled synapses increases with the progression of PCI, and is subsequently mediated by activated microglia to trim and engulf synapses, resulting in neuronal damage [115].

In the patient’s brain with AD, C3 mRNA and protein expression are up-regulated [116]. Similarly, in the PS19 model mice, C3 is also significantly up-regulated [117]. The expression and localization of C3 in the lateral geniculate nucleus (LGN) of the developing visual system are similar to that of C1q, both C1q and C3 deficiency have significant defects in synapse elimination [114, 118]. In the developing visual system, C3 selectively labels weak synapses, which are recognized and engulfed by microglia via CR3, followed by refinement of the remaining synaptic arbors, and disruption of the microglia-specific CR3/C3 signaling leads to persistent defects in synaptic connections and brain wiring [119]. In APP/PS1 transgenic model mice, a decrease in synaptic density in the hippocampus was observed by TEM [120]. In addition, blocking complement and downstream iC3b/CR3 signal transduction before Aβ accumulation can inhibit synaptic loss and prevent cognitive decline [121]. Complement C3-mediated abnormal synaptic pruning by microglia also induces depression-like behavior in mice with intestinal dysbacteriosis [122].

Sekar and colleagues found that the C4 expression levels are higher during the synaptic developmental stage in the dorsolateral geniculate nucleus and C4-deficient mice showed defects in synaptic elimination in the retinal generation system [123]. Similarly, patients with schizophrenia experience substantial loss of grey matter and dendritic spines during the onset of the disease. In co-cultured human iPSC-derived neurons and microglia, C4 can promote synaptic uptake by microglia [124]. Increased expression of C4-related genes as C4a induces greater phagocytosis of synapses by microglia as well as excessive synaptic refinement in the dorsal LGN [125]. Taken together, complement-mediated synaptic pruning plays a key role in NDDs. After stroke, the complement system is also a significant participant in synaptic pruning.

## The role of the complement system in synaptic pruning after stroke

The complement system has been recognized as an important player in synaptic pruning. Among them, complement proteins C1q, C3, and C4 play a key role in synaptic pruning, marking inappropriate synaptic connections between neurons that are cleared by phagocytic glia [50]. In recent studies, C1q [126], C3 [127], and C4 [128] were found to be significantly increased and they are closely related to synaptic pruning after stroke. Here, we summarize how these complements mediate synaptic pruning after stroke.

## The role of C1q in synaptic pruning

C1q, the classical complement initiator, is a macro secretory protein composed of C1qa, C1qb, and C1qc peptide chains [129, 130]. It regulates various immune cell responses, activate the body’s first line of defense against pathogens, and eliminate aging and apoptotic cells, playing a crucial role in regulating inflammatory response and maintaining autoimmune tolerance [131, 132]. In the normal brain, C1q is derived not only from microglia but also dendrites near synapses and abnormal mitochondria [133, 134].

**Figure 2.**
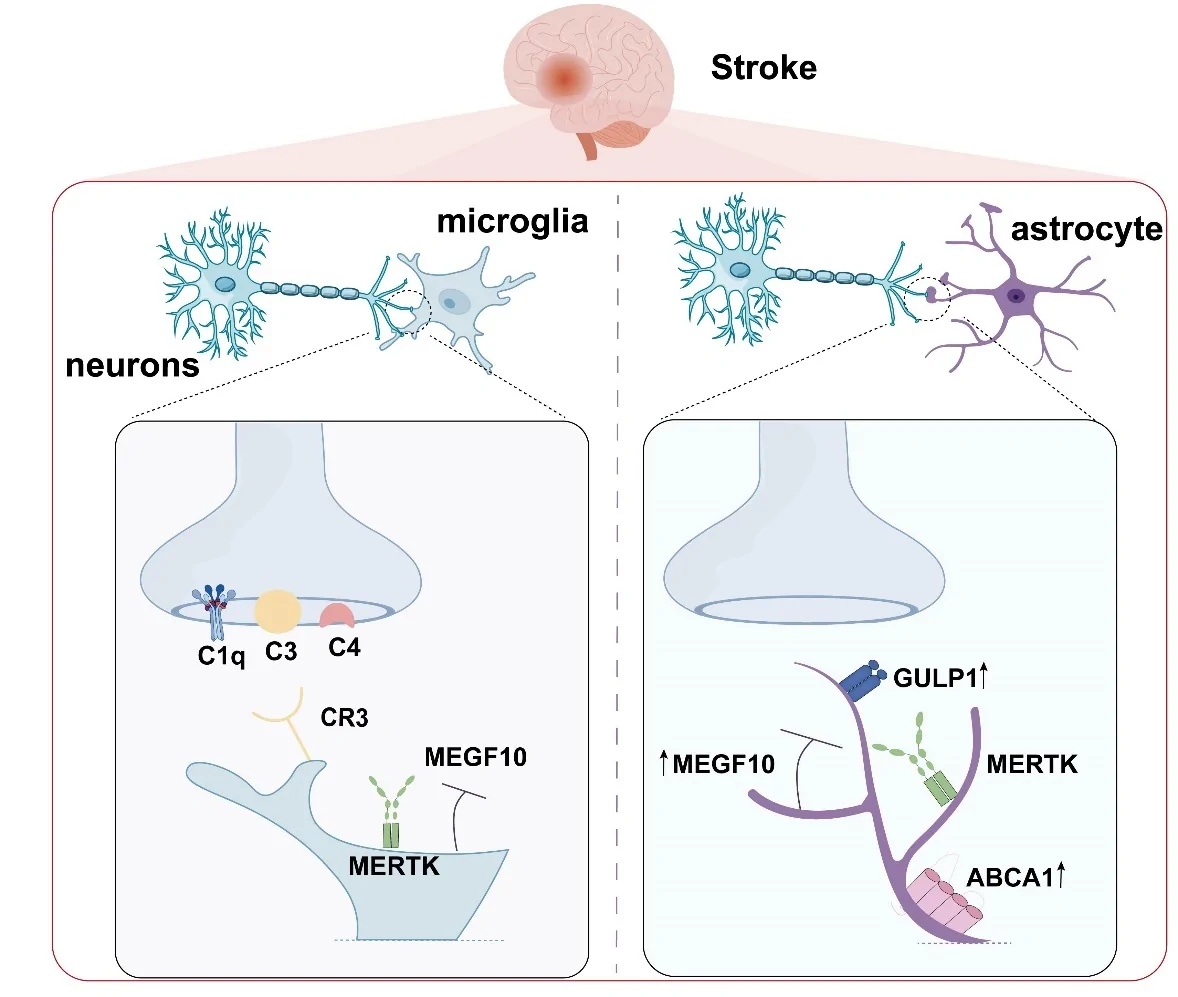
**Complement-mediated synaptic pruning in stroke**. After stroke, C1q, C3, and C4 may mark synapses, and the complement receptor CR3 on microglia can recognize C3 and engulf synapses. The expression of C1q in microglia can lead to synaptic loss. There are also MEGF10 and MERTK phagocytic receptors on microglia that can recognize phagocytose synapses. Astrocytes upregulate ABCA1 and its pathway molecules MEGF10 and GULP1, thereby transforming into phagocytic astrocytes that engulf degraded neuronal cell fragments and synapses.

**Figure 3.**
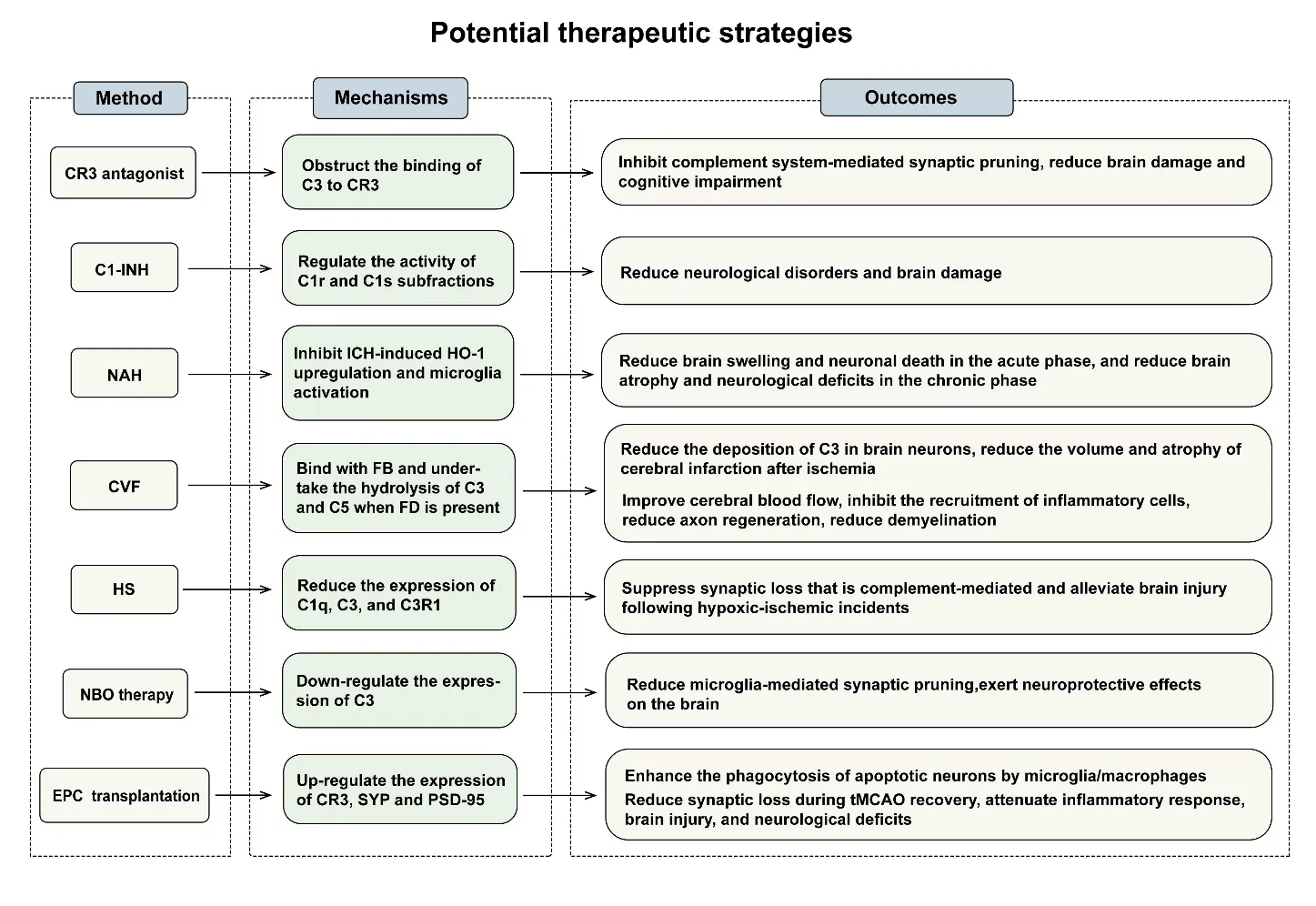
Treatments that may inhibit complement-mediated synaptic pruning.

C1q, involved in processes related to cell lysis and phagocytosis, contributes to hematoma regression [77]. In patients with AIS and ICH, serum C1q levels are significantly increased and positively correlated with clinical outcome [135, 136]. C1q deposition increased in the brain, and C1q-deficiency mice can inhibit neutrophil infiltration, oxidative damage and reduce cerebral infarction volume in the neonatal HI model mice [137, 138]. Furthermore, in autologous blood-induced ICH model mice, expression of C1q surrounding the hematoma significantly increased, mainly in microglia/macrophages, and peaked at day 7 after ICH [139]. In the model of rhesus monkeys cortical injury, Zhou et al [126] found that the excitatory and inhibitory synapses in the M1 and premotor cortices (PMC) of the injured peripheral motor cortex decreased, and the degree of reduction was related to the expression of C1q in microglia. Mesenchymal-derived extracellular vesicles (MSC-EVS) treatment can up-regulate the expression of anti-inflammatory C1q+ hypertrophic M1 microglia around the lesion to promote synaptic plasticity, thereby preventing excessive loss of synapses and chronic inflammation in PMC and protecting the synaptic cortical motor network and synaptic function (Fig. 2). In another study, Huang et al [140] observed a significant increase of C1q on the surface of neurons at day 1 of the MCAO model mice. They found that sialyl Lewis x (sLex)-glycosylated treatment can inhibit complement activation, reduce cerebral infarction tissue volume and nerve function defects. In the HI model mice, the expressions of C1q, C3, and C3R1 increased at day 3, and the PSD95 and SYP levels in the ischemic cortex decreased at day 3, 14 and 28 after injury. HS treatment significantly reduced the expression of C1q, C3 and C3R1, and restored PSD95 and SYP expression, reversing neurological dysfunction (Fig. 3) [106].

Therefore, inhibiting C1q can reduce the phagocytic effect of microglia on synapses, thereby alleviating brain injury and improving neurological dysfunction. These findings highlight the role of C1q in post-stroke synaptic pruning and provide potential therapeutic strategies to alleviate post-stroke brain injury.

## The role of C3 in synaptic pruning

C3, the most abundant and central complement protein in the blood, is composed of α and β chains and covalently linked by disulfide bonds and is mainly synthesized by macrophages and the liver [141, 142]. It is well known that the activation of the complement cascade reaction and MAC formation depend on the cleavage of the C3 component [102]. C3 convertase is a highly specific protein enzyme that hydrolytically cleaves the C3, which generates the primary inflammatory mediator C3a and the main opsonin molecule C3b [143]. C3a can attract immune cells by interacting with C3aR, regulate the immune response, and mediate downstream inflammatory responses [144]. C3a activates the phosphorylation of MAPK and nuclear factor-κB (NF-κB) in vitro, and up-regulates the expression of vascular cell adhesion molecule 1 (VCAM-1) and intercellular cell adhesion molecule-1 (ICAM-1) in primary brain endothelial cells. Depletion of C3 and C3aR inhibited the expression of VCAM-1 and E-selectin and decreased the recruitment of leukocytes into the brain [145]. Clinical studies have demonstrated that plasma C3 levels are elevated in patients with stroke [146, 147]. The plasma C3 level is associated with the prognosis of IS at 3 months post-stroke [148], and positively correlated with clinical outcome [139, 147]. In the MCAO model mice, C3 deficient have significantly reduced infiltrating granulocytes and oxidative stress levels [85]. Treatment with the antioxidant N-tert-butyl-α-phenylnitrone (PBN) can inhibit the expression of C3, thereby reducing inflammatory response, infarct volume, and neurological deficits in the tMCAO model mice [149]. In the autologous blood-induced ICH model male mice, Yang et al [150] observed that C3 deficient has less brain edema, lower heme oxygenase-1 (HMOX1) levels, fewer activated microglia and infiltrated neutrophil infiltration in perihematoma than wild-type mice, ultimately leading to a reduction of brain injury. Zheng et al [139] revealed that CR3-positive microglia increased after ICH. The use of CR3 agonist Leukadherin-1 (LA-1) increases the ROS production, promotes neuronal cell death and aggravates neurological deficits, which up-regulates the expression of the CD163/HO-1 pathway and accelerates hematoma regression. Furthermore, oral minocycline, a broad-spectrum tetracycline, has a neuroprotective effect after ICH, which partly inhibits the C1q/C3-CR3 signaling after ICH.

In the rat model of bilateral common carotid artery occlusion (BCCAO), Zhang and his colleagues found that the levels of C3 and C3aR increased, which promoted microglia to engulf the myelin sheath and aggravated white matter damage. After administration of C3aR antagonist SB290157, the number of microglia adhered to the myelin sheath was significantly reduced, the white matter damage in the striatum was alleviated, and cognitive dysfunction was improved (Fig. 2) [127]. In the MicroE model mice, there was an increase in perilesion microglia hyperactivity and synaptic density in microglia (SV-2). Combination of B4Crry with tPA treatment of reperfusion mice was found to prevent the long-term deposition of C3d in perilesion synapses, reduce microglia proliferation and synaptic uptake, enhance cognitive function [67]. In the tMCAO model mice, the transplantation of endothelial progenitor cells (EPC), derived from hematopoietic stem cells in the bone marrow [151], amplify the phagocytic activity of microglia/macrophages towards apoptotic neurons through up-regulating the expression of CR3. At day 14 after tMCAO, EPC transplantation reduces the expression of C3aR, attenuates inflammatory response, up-regulates the expression of SYP and PSD95, restores neurological function (Fig. 3) [107, 152]. Since the brain is sensitive to hypoxia, NBO is considered to be a neuroprotective therapy for stroke. In collagenase-induced ICH model mice, Wu et al [68] found that NBO exerts neuroprotective effects on the brain by down-regulating C3 expression in microglia and reducing microglia-mediated synaptic pruning (Figure 3). Hence, C3 induces phagocytosis in microglia thereby mediating synaptic pruning. Inhibition phagocytosis via the C3-C3R pathway in microglia attenuates brain injury and improves neurological deficits.

## The role of C4 in synaptic pruning

C4, a disulfide-linked triple chain glycoprotein composed of α (95kDa), β (75kDa), and γ (30kDa) chains, is a subunit of C3 and C5 convertase and plays an integral role in activating the classical and lectin complement pathways, which is involved in recognition and elimination of microorganism [153]. MASP 2, derived from C1s activated by the CP or the LP, cleans the amino-terminal portion of α chain at a single site of C4 to generate C4a (9kDa) and C4b (195kDa) [154].

In a recent study, Wu et al [128] revealed that plasma C4 levels significantly increased and were closely linked to hematoma volume and clinical outcome after ICH. In schizophrenia like conditions, the role of C4 in synaptic pruning is significant. It interacts with synaptic markers VGLUT1/2 and PSD95 orientation [123]. Additionally, overexpression of the schizophrenia-related gene C4A promotes excessive synaptic loss and behavioral changes through synaptic phagocytosis of microglia in the mouse model [125]. Therefore, C4 may lead to brain damage and behavioral abnormalities by regulating synaptic phagocytosis dependent on microglia. However, the precise role of C4-mediated phagocytosis of synapses after a stroke warrants further investigation.

## Strategies for inhibiting complement mediated synaptic pruning

Following a stroke, the complement cascade is triggered, leading to the accumulation of C1q and C3 around neurons. Microglia then engulf synapses by attaching to CR3 on the synaptic surface, resulting in synaptic loss and neuronal fatality [127, 140]. Therefore, it is speculated that hindering the excessive activation of the complement system may impede synaptic pruning, augment neurological prognosis, and accelerate the recovery of stroke patients.

First of all, CR3 antagonists can block the binding of C3 to CR3 and inhibit the synaptic pruning, thereby alleviating brain injury and cognitive impairment. Complement consumption agents and inhibitors can also be used to reduce synaptic pruning. Then the complement inhibitor C1-INH regulates the activity of C1r and C1s subfractions [155]. In the MCAO model mice, it was found that the application of C1-INH treatment reduces neurological disorders and brain damage [156]. Similarly, in autologous blood-induced ICH model rats, co-injection of a complement inhibitor N-acetyl heparin (NAH) could inhibit ICH-induced HO-1 upregulation and microglia activation, reduce brain swelling and neuronal death in the acute phase, and attenuate brain atrophy and neurological deficits in the chronic phase (Fig. 3) [157].

Cobra venom factor (CVF), a three-stranded 149 kDa non-toxic glycoprotein linked by disulfide bonds from cobra venom, is a structural homolog of C3b. It can bind to factor B, and then factor B is cleaved by factor D to produce the C3/5 convertase CVFBb, which hydrolyzes and depletes C3 and C5, thereby inhibiting the activation of classical and alternative complement pathway [158, 159]. In HI neonatal model mice using CVF, the deposition of C3 decreased in brain neurons [160], and in adult and neonatal MCAO model in rats, the use of CVF significantly reduced post-ischemic cerebral infarct volume and atrophy [161]. Simultaneously, systemic depletion of the complement component using CVF may enhance cerebral blood flow, suppress the recruitment of inflammatory cells and decrease axonal regeneration and demyelination (Fig. 3) [162-164]. As a consequence, C3-depleting agents can be utilized to diminish the amount of C3, which in turn could hinder the C3-C3aR pathway and reduce the phagocytosis of microglia. This would result in the inhibition of complement-mediated synaptic pruning, mitigating stroke-induced brain damage and cognitive impairment. Therefore, it is very important to use drugs to inhibit the overactivation of the complement system, the production of complement and the synaptic pruning mediated by the complement system, which may help to improve brain injury and promote early recovery of neurological function.

## Perspective for post-stroke management

Post-stroke management is based on well-organized active care, close neuromonitoring by well-trained staff, and early detection and management of complications [165]. Cognitive impairment can be treated by cognitive training, physical activity and medication after stroke [166]. Effective rehabilitation treatment after stroke can accelerate the rehabilitation process, reduce functional disability and save social resources. In addition, it is necessary to explore a wide range of alternative long-term sustainable interventions to improve neurological function, including new drug therapy, stem cell therapy, rehabilitation therapy, and to support stroke survivors in participating in meaningful activities and addressing post-stroke challenges.

## Advances in therapeutic strategies for stroke

During current basic research and clinical practice, some drugs have shown to have certain therapeutic effect. In a prospective, multicenter, phase IV study (RESK study), human urinary kallikrein (HUK) improved neurological function [167], and reduced the risk of PSCI at 3 months in AIS patients [168]. The relevant mechanism involves in enhancing angiogenesis and cerebral perfusion [169]. A novel peptide-templated manganese dioxide nanozyme (PNzyme/MnO_2_) may serve as a novel therapeutic agent to improve the efficacy and prevent secondary thrombosis during the treatment of IS [170]. A previous clinical study showed that sovateltide improved neurological function in AIS patients after day 90 of treatment [171]. An ongoing multicenter, randomized, double-blind, parallel, placebo-controlled phase III study was designed to evaluate the safety and efficacy of sovateltide in patients with AIS (NCT05691244). Pentoxifylline (PTX), a non-selective phosphodiesterase inhibitor, reduced ischemic white matter damage by mediate microglia to clear myelin [172]. The effect of PTX on cognitive impairment after IS (NCT06344390) is an ongoing clinical study.

There are also some drugs that exert neuroprotective effects by regulating synaptic function. Folic acid, a water-soluble B vitamin, significantly reduces the risk of stroke by 10% using a largest randomized controlled trials meta-analysis [173]. Folic acid diet increases synaptic proteins expression (such as SYP and PSD95), synaptic density, and improves the cognitive ability in MCAO/R rats [174]. Long-term application of oleanolic acid (OA), a natural pentacyclic triterpenoid, inhibits astrocyte and microglia activation, enhances synaptic-related proteins expression, and promotes synaptic connection and nerve regeneration [175]. Drugs such as agrin [176], salvia miltiorrhiza [177], and rhynchophylline [178] can improve synaptic function and promote the recovery of neurological function after stroke.

The brain is highly sensitive to hypoxia, and clinical trials have shown that NBO combined with endovascular treatment (EVT) can effectively improve the functional outcome of patients with acute anterior circulation stroke within 6 hours after stroke, reduce mortality and infarct volume [179, 180]. Stem cell-based therapy is one of the novel strategies for treating stroke and is involved in neuronal repair and remodeling following IS [181-183]. In randomized controlled trials, mesenchymal stem cells (MSCs) have been shown to improve neuroplasticity and promote motor recovery after stroke [184, 185]. Similarly, human umbilical cord MSCs overexpressing HO-1 [186], human-induced pluripotent stem cell-derived mesenchymal stem cells (hiPS-MSC-EVs) [187] and MSC-EVs [188] also showed therapeutic effects in animal models.

## Rehabilitation therapy to improve neuroplasticity

After stroke, the damage disrupts the integrity of neural circuits and brain networks, leading to further impairment of broader structure and function. Neuroplasticity includes dendritic and dendritic spine remodeling, axonal sprouting, synaptic formation and synaptogenesis. It is the basis for the rehabilitation and neurological recovery of stroke patients [189]. Exercise is widely regarded as an effective and feasible rehabilitation strategy, which helps improve cognitive and motor function recovery by promoting neural plasticity. A study found that exercise enhances the postsynaptic excitability and neuronal activity of the layer 5 pyramidal neuron (L5PRN) in the motor cortex and enhances the formation of axon myelin [190]. Exercise increases the synaptic plasticity-related proteins expression by regulating the content of exosomes, thereby promoting the recovery of motor function in the MCAO rat model [191]. For patients with cerebrovascular accident (CVA), exercise combined with carbohydrate/protein supplementation can maximize the effectiveness of exercise rehabilitation [192]. Acupuncture (AC) therapy has been shown to increase the expression of brain-derived neurotrophic factor (BDNF) and SYN in the ipsilateral hippocampus and promote synaptic plasticity [193]. Similarly, some previous clinical studies have shown that AC may be effective in the treatment of PSCI and further investigation is still needed to provide powerful evidence for AC and moxibustion treatment of PSCI [194, 195]. Electroacupuncture (EA) promotes nerve regeneration and enhances nerve plasticity by increasing the expression of BDNF [196]. Similarly, noninvasive brain stimulation (NIBS) is a popular neuromodulation rehabilitation technique that can improve clinical function by regulating the excitability of the corresponding neurons [197]. Among NIBS, repetitive transcranial magnetic stimulation (rTMS) is the most widely studied and applied which can improve stroke dyskinesia and has a positive impact on the functional recovery of patients with stroke [198]. Yin et al [199] performed 20 sessions of 10 HZ rTMS of the left dorsolateral prefrontal cortex in patients with PSCI and showed that rTMS had significant improvements in cognitive function and daily activities. To sum up, comprehensive and multidisciplinary methods combined with drug and non-drug therapy play an important role in the rehabilitation after stroke. By using these methods, we can provide patients with more comprehensive and effective rehabilitation treatment to help them recover at an early date.

## Conclusion and prospect

Stroke patients not only face challenges with physical function, but may also encounter complications that affect their functional outcomes and recovery process. Those patients may experience psychological stress and social adaptation difficulties, such as depression, anxiety, social isolation and other common problems. However, these problems are often not given enough attention and timely intervention, which will have a negative impact on the quality of life of stroke patients. Stroke rehabilitation begins during acute hospital care and runs through the entire life cycle of patients with stroke [200]. By in-depth study of the patient’s needs and translating these needs into specific treatment options and technical applications, the challenges in the patient’s rehabilitation process can be more effectively addressed. In view of the significant role of complement in mediating synaptic pruning in NDDs, as well as the overactivation of complement and the occurrence of synaptic phagocytosis by glial cells after stroke, it seems that there are indications that PSCI is associated with synaptic loss. This article summarizes the various roles of complement after stroke and the related pathological mechanisms of brain injury caused by synaptic pruning through the complement system.

Microglia-mediated phagocytosis is typically advantageous for brain recuperation and is necessary for the elimination of synaptic waste. However, microglial synapse phagocytosis can also be detrimental since excessive synaptic consumption can impair brain function. In cases of stroke, there is a possible occurrence of immune molecules acting as “eat me” signals to label synapses. CR3, expressed on microglia, recognizes C3, leading to synaptic loss and neuronal death in the brain, causing neurobehavioral abnormalities and brain damage. It is known that EPC transplantation and HS treatment can inhibit synaptic loss, alleviate brain injury, and restore neurological function. Therefore, more attention should be paid to how to inhibit complement-mediated synaptic pruning after stroke.

The establishment of a complement system plays a crucial role in synaptic loss and cognitive impairment. However, it is worth noting that research on how glia mediates synaptic pruning through the complement system is limited after stroke. Due to the limitations of some drugs found in stroke treatment, can we achieve better results by inhibiting synaptic pruning. Hence, it is crucial to further examine other mechanisms by which the complement system mediates synaptic pruning in the future. Additionally, researchers should investigate other complement or substances involved in complement-mediated synaptic pruning and formulate safe and efficient drugs or novel therapeutic approaches to inhibit synaptic pruning. By doing so, new therapeutic targets can be explored to alleviate brain damage and slow down cognitive impairment, leading to a decreased burden in clinical practice.
